# Supplementary material for: Exploring the Prognostic Role of Neurofilaments and SEMA3A in Multiple Sclerosis Progression
Source: Int J Mol Sci. 2025 Sep 8;26(17):8750. doi: 10.3390/ijms26178750 (PMC12429374; doi:10.3390/ijms26178750)
Supplement: Supplementary file 1 [file ijms-26-08750-s001.zip › ijms-3796117-supplementary.pdf]

**Supplementary materials**

**Supplementary Table S1.** Multivariate regression analysis to validate differences between RR MS and SPMS groups in studied biomarkers

| Outcome (*Y*) | Estimated regression coefficients | | | |
|---|---|---|---|---|
| | Exploratory variable (*X*) | Estimate | Std. Error | P-value |
| Progranulin | (Intercept) | 5.522020 | 2.154864 | 0.0117 |
| | group - SPMS | -2.178006 | 1.437986 | 0.1326 |
| | age | 0.006563 | 0.048003 | 0.8915 |
| | Disease Duration | 0.044998 | 0.055849 | 0.4221 |
| IL-6 | (Intercept) | 335.676 | 228.578 | 0.145 |
| | group - SPMS | 82.342 | 152.535 | 0.590 |
| | age | 1.725 | 5.092 | 0.735 |
| | Disease Duration | -2.634 | 5.924 | 0.657 |
| SEMA3A | (Intercept) | 8.0884 | 5.3871 | 0.136 |
| | group - SPMS | 3.8907 | 3.5949 | 0.281 |
| | age | 0.1566 | 0.1200 | 0.195 |
| | Disease Duration | -0.1922 | 0.1396 | 0.171 |
| Neurofilaments | (Intercept) | 44.04740 | 16.15655 | 0.00742 |
| | group - SPMS | 4.74302 | 10.78160 | 0.66083 |
| | age | 0.02904 | 0.35992 | 0.93584 |
| | Disease Duration | 0.21301 | 0.41874 | 0.61195 |

Legend: Multivariate regression models was fitted to validate differences between groups: Biomarker (*Y*) = group (reference group = RR MS) + age + disease duration.

**Supplementary Table S2.** Multivariate correlation coefficients for RRMS (above diagonal) and SPMS patients (below diagonal)

| | **Progranulin** | **IL6** | **SEMA3A** | **NEUROFILAMENTS** | **EDSS** |
|---|---|---|---|---|---|
| **Progranulin** | 1 (0) | 0.09 | 0.14 | 0.08 | 0.09 |
| **IL6** | 0.39 | 1 (0) | 0.05 | 0.14 | 0.13 |
| **SEMA3A** | 0.26 | 0.20 | 1 (0) | 0.25 | 0.15 |
| **NEUROFILAMENTS** | 0.32 | 0.16 | 0.44 | 1 (0) | 0.21 |
| **EDSS** | 0.21 | 0.17 | 0.32 | 0.12 | 1 (0) |

Legend: Multiple correlation coefficients for RRMS are reported (in brackets) above the diagonal and for SPMS patients below the diagonal. The coefficients were calculated to validate the correlations after adjustment for confounding factors age and MS duration.
